# Transactions of the American Medical Association

**Published:** 1852-09

**Authors:** 


*Transactions of the American Medical Association.* — We copy the following notice from the Stethoscope, edited by Dr. Gooch, one of the Secretaries of the Am. Med. Association:

"The readers of the Stethoscope are hereby informed that the volume of the Transactions of the American Medical Association, containing all the interesting papers and reports presented at the meeting held in Richmond in May last, and making about 1000 pages, will soon be issued. Members, or institutions represented in that body, will be furnished with one copy for $3, or two copies for $5, *if the amount is remitted to us, or to Dr. D. Francis Condie*, at Philadelphia, *previous to the* 1*st September.* After that time the price of the volume will be *five dollars.* We advise those desiring to be prompt."

We are informed in a letter from Dr. Condie, that the volume will be ready for distribution early in October.

